# Prevention and intervention strategies to check increasing violence against Healthcare Facilities and Healthcare Professionals

**DOI:** 10.12669/pjms.321.9658

**Published:** 2016

**Authors:** Shaukat Ali Jawaid

International Committee of the Red Cross (ICRC) initiated a multicenter research study on violence against healthcare in Karachi some time ago and its report has now been published. Apart from ICRC, Jinnah Postgraduate Medical Center, Ziauddin University and Jinnah Sindh Medical University had participated in this study and the report was formally released at a meeting in Karachi on November 13^th^ 2015 which was largely attended by the different stakeholders.

Principal investigator Prof. Lubna Baig Dean, APPNA institute of Public Health who has now also been appointed Pro Vice Chancellor of JSMU and his team has done a commendable job in pointing out the reasons for violence against healthcare professionals (HCPs) and Healthcare facilities (HCFs). The most important reasons highlighted in this study include unreasonable expectations (56.1%), unexpected outcome (42.6%), communication failure (55%), and human error (53.7%) besides substandard care (35%). Poor quality of service provided by the healthcare facilities besides low capacity of healthcare professionals are also reported to have contributed to these violent incidents. The report has also pinpointed that a vast majority of the perpetrators of this violence were the patient’s attendants.^1^ They collected the data from hospitals, ambulance service and Non-Governmental Organizations. The report is based on eight hundred twenty two questionnaires which were received and analyzed. The report further highlights that majority of the violent incidents 41.8% occurred in Accidents and Emergency followed by 39.4% in emergency obstetrics, 13.6% in wards and majority of the violent incidents were reported from public hospitals.^1^

In fact increasing commercialization and corruption along with wide spread unethical practices by the medical profession has also lead to increased violent incidents.^2^ Numerous studies from China have also highlighted increasing incidents of violence against healthcare professionals which unfortunately lead to death of some doctors and nurses by the patient’s relatives. According to study by Hongzing Yu et al from China poor quality of services, increased awareness of the patients about their rights and their willingness to take legal action, negative media reports about hospitals and doctors, out of pocket medical expenditures by the patients, lack of trust in doctors and hospitals were reported to be some of the causative factors.^3^-^6^

Violent incidents against healthcare professionals have also been reported in other countries in this region i.e. India, Bangladesh and Pakistan by Ali Khawaja and Hira Irfan^7^ Nazish Imran et al,^8^ Mirza NM^9^ Madhok from India^10^ and HAM Nazmal Ahsan from Bangladesh.^11^ A critical analysis of these studies also reveals that most of the triggers for violence in all these studies are also common such as lack of communication between doctors and the patient, unexpected expectations and unexpected outcome, poor image of the medical profession, sub-standard quality of care besides insufficient security for doctors. A wide gap exists between the patients’ expectations and the reality and all this has certainly decreased self-esteem of the doctors as well. These studies have also similar findings as regards the place of occurrence of these incidents which most often has been in the Emergency and Accidents Departments.

Violence against healthcare professionals has also been reported from Saudi Arabia, Kuwait and Australia.^12^-^14^ Hence if on one hand these studies highlight the reasons for this violent incidents, they also point towards effective intervention strategies which if undertaken can go a long way in checking and minimizing the occurrence of such incidents. Hence, one hopes that the concerned authorities in Pakistan as well as in other countries facing such a situation will undertake effective administrative and other measures. We had earlier suggested various measures which included improving communication skills of healthcare professionals and eliminating the communication gap between the healthcare professionals and the patient’s as well as their attendants, involving patients and their family members in decision making regarding management of their diseases, providing security at healthcare facilities with proper boundary walls with adequate security system and controlled exit points, allowing patients attendants only during visiting hours, posting experienced staff in the Emergency, appointing liaison officer by healthcare facilities to speak to the media, banning entry of media personnel particularly from the electronic media to HCFs without permission, improving the services, adequate explanation of likely complications of surgical procedures, avoiding negligence and giving 100% assurance of cure, avoiding over confidence by the HCPs, initiating self-monitoring of the healthcare professionals by the respective professional specialty organizations in collaboration with the institutions where they work and above all ensuring ethical medical practice.^2^ ICRC report has also come up with useful recommendations for institutions, society at large, social reforms, role of stake holders like merit based culture, doing away with VIP culture, training of healthcare professionals, provision of adequate facilities, role of law enforcing agencies as well as professional bodies.^1^ These suggestions and recommendations needs to be implemented, an area where we in Pakistan have a very poor track record. Without ensuring their implementation, the ICRC report will also prove to be yet another futile exercise.

Media should also follow some code of ethics while reporting such events because continuous negative reporting will eventually go against the interests of the patients whose interest they wish to safeguard. Already healthcare professionals are now reluctant to handle serious cases with the result that many precious lives which they could have saved are being lost. Serious cases are often referred to tertiary care facilities. Transporting these patients takes some time which could make a difference in the eventual outcome of such cases. Government authorities also need to wake up and initiate steps to implement some of the above and other recommendations by the research team of ICRC which in addition to Prof. Lubna Baig included Prof. Kamran Hameed, Dr. Kausar S. Khan, Dr. Seemin Jamali and Dr. Syeda Kausar Ali. In many cases no additional funding is required but just ensuring judicious use of existing facilities and resources and administrative measures will go a long way in improving the situation to a great extent immediately while long term plans can be prepared and implemented later on.
